# Successful pregnancy of an SMA type 3 sitter on Nusinersen therapy - a case report

**DOI:** 10.1186/s12883-024-04005-3

**Published:** 2025-01-04

**Authors:** Miriam Hiebeler, Simone Thiele, Maggie C. Walter

**Affiliations:** 1 Department of Neurology, Friedrich-Baur-Institute, Ludwig-Maximilians-University of Munich, Munich, Germany; 2 Department of Neurology, Friedrich-Baur-Institute, Ludwig-Maximilians-University of Munich, Ziemssenstrasse 1, 80336 Munich, Germany

**Keywords:** Nusinersen, Pregnancy, Spinal muscular atrophy, Disease-modifying drugs

## Abstract

**Background:**

Due to improved treatment options, more SMA patients reach childbearing age. Currently, limited data on pregnant SMA patients is available, especially in relation to disease-modifying therapies (DMT). This case report helps to elucidate new approaches for future guidelines in the management of pregnancy and SMA.

**Case Report:**

A 33-year-old wheelchair-bound patient with SMA type 3 (sitter) became pregnant following 36 months of Nusinersen treatment. The last dose was administered in the third gestational week. After pregnancy was confirmed, therapy was stopped immediately. A healthy child was born in the 34th gestational week by caesarean section. After a short nursing period, Nusinersen was restarted 6 weeks after the expected gestational date. At this time, the patient reported deteriorated motor functions, which stabilized at a lower level compared to pre-pregnancy in the 2-year follow-up, despite restarting Nusinersen treatment.

**Discussion:**

So far, only few cases of successful pregnancies of SMA patients on DMT have been reported. In natural history, the majority of patients experienced an increased deterioration of motor function while fetal outcome was not impaired. Our case shows that although Nusinersen treatment was applied in the third gestational week prior to proof of pregnancy, outcome was positive for mother and child. Future studies will have to determine whether ongoing treatment with Nusinersen during pregnancy should be recommended.

## Introduction

Spinal muscular atrophy is an autosomal recessive inherited disease leading to loss of motor neurons in the brainstem and spinal cord. Clinically, progressive muscle weakness and muscle atrophy are the main symptoms. During the natural course of the disease, failure of respiratory muscles will occur together with an increased loss of motor functions. Physiotherapeutic assessments such as HFMSE (Hammersmith Functional Motor Scale-Expanded), RULM (Revised Upper Limb Module) and 6MWT (6 min Walk Test) are used to monitor the motor functions of the adult patients [1–5].

Improved therapeutic options for spinal muscular atrophy have enabled the majority of patients to reach childbearing age [6]. To date, only few data and guidelines on SMA patients and pregnancy are available. While an increased rate of caesarean sections and premature labour have been reported, the risk of maternal and fetal complications does not appear to be increased in pregnant SMA patients compared to the general population [7–9].

For adults, two different disease modifying drugs are available, Nusinersen and Risdiplam. Nusinersen is an intrathecally administered antisense oligonucleotide altering the splicing of SMN2 mRNA, hereby leading to increased levels of the SMN2 protein. The clinical benefit of Nusinersen for adult patients with SMA has been demonstrated in numerous studies and real-world evidence reports showing improvement and preservation of motor function [7–9].

Risdiplam is a small molecule, systemically active oral splicing enhancer that increases the expression of the functional SMN protein by modulating SMN2 splicing. In three clinical studies with RG7916: SUNFISH (SMA 2 and SMA 3), FIREFISH (SMA 1) and JEWELFISH (pre-treated patients with SMA 2 and SMA 3), Risdiplam showed a significant clinical benefit in infantile SMA, less pronounced in later-onset SMA [10–13].

Since June 2020, conditional approval in Europe of the gene replacement therapy onasemnogen abeparvovec for patients with a proven mutation of the SMN1 gene and a clinical diagnosis of SMA 1 regardless of the copy number of the SMN2 gene, or for SMA patients with a mutation of the SMN1 gene with up to 3 SMN2 copies regardless of the SMA type. An age and/or weight restriction has not yet been established, but the drug is only used in newborns and young children according to the 2024 European consensus statement on gene therapy for spinal muscular atrophy [14]. Since the risk of gene therapy increases with the dose administered and since the dose is proportional with the weight and age, heavier and older patients should be treated very cautiously as the data available in these patients are very scarce.

However, outcome of the individual patients with either drug does not depend on SMA type or age, but mainly on preserved motor function prior to onset of treatment.

To date, there is only very limited experience with the use of Nusinersen in pregnant women. According to the Information for healthcare professionals, animal studies have not shown any evidence of direct or indirect adverse health effects with regard to reproductive toxicity. Nusinersen does not cross the blood-brain barrier to any relevant extent, but for precautionary reasons the use of Nusinersen should be avoided during pregnancy and breastfeeding [15].

There is already published experience with pregnancies that have occurred during treatment with Nusinersen [16, 17]. Therapy should be started after a Nusinersen break due to pregnancy/breastfeeding in accordance with the dosing regimen specified in the Nusinersen Information for healthcare professionals if the dose is delayed or omitted [18]; pharmacokinetic studies have shown that an additional dose should be given for each omitted maintenance dose in order to achieve steady-state exposure in the cerebrospinal fluid [19].

There is a fertility risk for Risdiplam in men; a Risdiplam break of at least four months before conception is recommended according to the information for healthcare professionals; strategies for maintaining fertility (sperm asservation) should be discussed with male patients of reproductive age before starting treatment. In women, attention is drawn to possible embryofetal toxicity, which has been shown in animal studies; a risk-diploma break of at least one month is recommended before pregnancy occurs. Safe contraception is generally obligatory with Risdiplam; the pregnancy status of patients capable of reproduction should be checked before starting treatment with Risdiplam. It is also recommended not to breastfeed during treatment with Risdiplam [20].

While extensive data on pregnant SMA patients from the pre-treatment era is not available, even less is known with regard to pregnancy and DMT. Here we present a wheelchair-bound patient with SMA type 3 (sitter), who became pregnant during DMT with Nusinersen. We aim to contribute new approaches for future guidelines in the management of pregnant SMA patients on DMT.

## Case report

We report on a 33-year-old female patient with confirmed SMA type 3b (sitter) with homozygous deletion of *SMN1* gene exon 7 and 8; 3 *SMN2* copies have been identified. After reaching all motor milestones timely up to the age of two, first visible symptoms appeared in form of weakness when climbing stairs. Early motor development was unremarkable otherwise; sitting was possible at six months and independent walking at twelve months. The symptoms progressively worsened until the patient was finally wheelchair bound from the age of thirteen, when walking and standing were no longer possible. Difficulties with tasks involving raising the arms above the head had already been reported for several years.

Treatment with Nusinersen was initiated at the age of twenty-seven. Clinical neurological findings at that time included tetraparesis (arm abduction 4/5 both, hip flexion 2/5 both) as well as areflexia and contractures of both knee joints and the Achilles tendons. Respiratory muscles were not affected. The baseline physio assessments yielded 23 out of 66 points in HFMSE, and 31 out of 37 points in RULM on both sides.

The patient received Nusinersen for 36 months according to the usual intrathecal regimen (four loading doses, then every four months) without any relevant side effects. During treatment, there was clinically meaningful improvement: HFMSE increased 8 points (from 23 to 31 points), and RULM 1 point (from 31 to 32 points) bilaterally. However, hand grip slightly decreased over time, while vital capacity remained largely stable (see Table 1).

**Table 1:** Scores of our patient over time

Nusinersen – Visit-#	HFMSE (66)	RULM-*r* (37)	RULM-l (37)	VC (l)	HG-*r* (kg)	HG-l (kg)	MIP (cmH_2_O)	MEP (cmH_2_O)
**1** Baseline	23	31	30	3,22	-	-	-	-
2	-	-	-	-	-	-	-	-
3	-	-	-	-	-	-	-	-
4	33	30	30	3,28	-	-	-	-
5	30	31	31	3,32	-	-	-	-
6	-	-	-	3,32	-	-	-	-
7	33	31	31	3,49	-	-	121	71
**8** 18 months therapy	33	30	30	3,50	11	10	114	72
9	33	30	31	3,35	-	-	110	63
10	32	32	32	3,38	11	10	115	76
11	28	32	31	3,35	10	9	142	78
12	32	32	33	3,33	9	8	142	74
**13** 38 months therapy	31	32	32	3,20	10	8	123	70
25th week of pregnancy	30	32	32	3,32	9	8	129	64
14	32	28	28	3,22	11	10	117	56
15	-	-	-	-	-	-	-	-
**16** 11 months after interruption	30	29	29	3,20	12	10	140	62
17	30	29	29	-	12	10	145	65
18	26	25	28	-	11	10	123	67
19	27	27	28	3,26	12	11	139	72
20	26	27	27	-	12	11	142	62

*HFMSE* - expanded Hammersmith Functional Motor Scale; *RULM-r/l* - Revised Upper Limb Module for SMA, right or left side; HG-r/l – Handgrip right or left side; MIP – maximum inspiratory pressure - maximum expiratory pressure; VC - Vital capacity

**Fig. 1:**
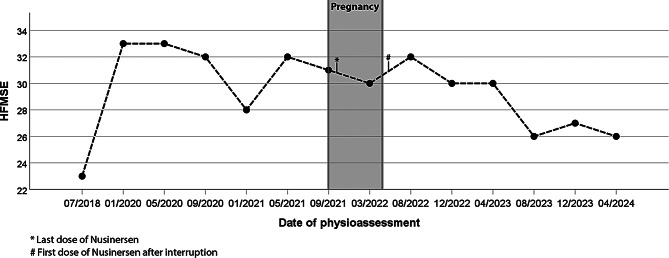
RULM over time

**Fig. 2:**
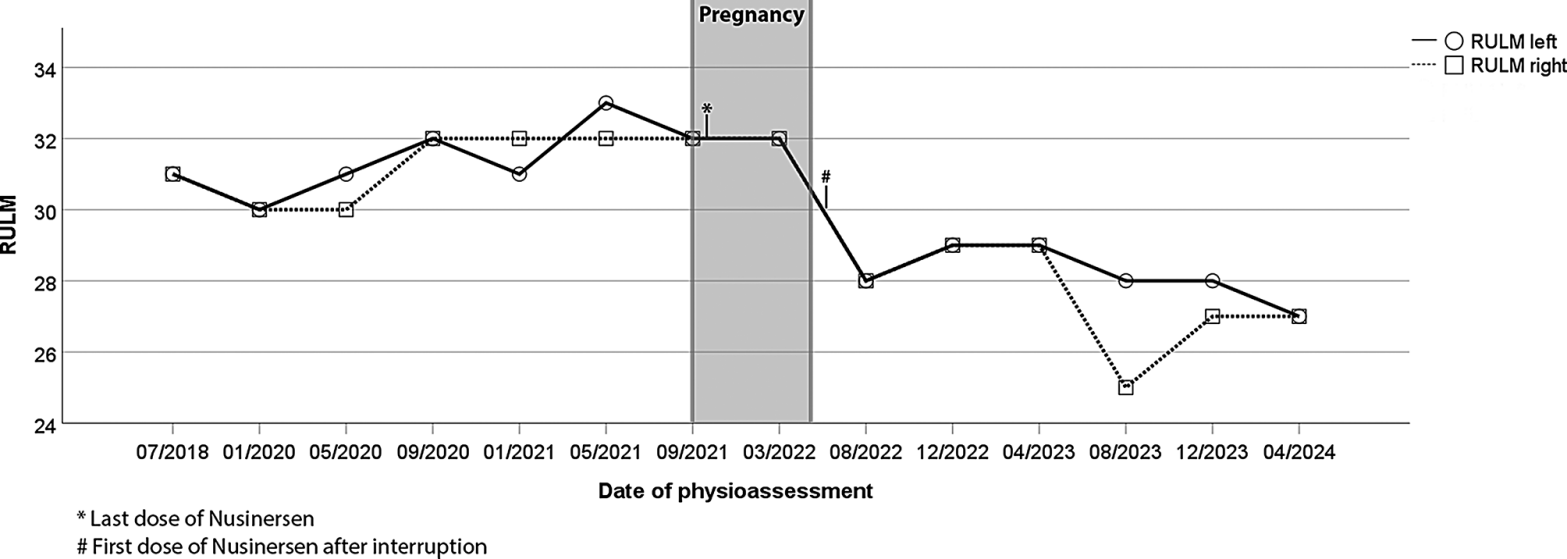
HFMSE over time

At the age of thirty, the patient became pregnant whilst treated with Nusinersen, which was last administered for the last time during the third week of pregnancy. With the confirmation of pregnancy, treatment with Nusinersen was paused after a total of thirteen doses. In the 25th week of pregnancy, physio assessments were performed. HFMSE revealed minimal deterioration of one point (31 to 30), while RULM showed stable values. Scores are shown in Figs. 1 and 2.

Birth was given during the 34th week of pregnancy by caesarean section due to maternal HELLP syndrome. Elevated blood pressure values of the patient had already been seen beforehand. At birth, the daughter was normotrophic for gestational age. The APGAR score was 8/8/9 with complete lung maturity. During the next two years follow-up, the child showed completely normal motor development.

Six weeks after the expected gestational date, after a short breast-feeding period, treatment with Nusinersen was restarted with another two loading doses fourteen days apart. At this time, the patient reported a subjective deterioration in motor function, particularly in the arms, which could also be objectified using physio assessments (HFMSE stable, RULM 28 bilaterally). Detoriation of upper arm abduction was detected by manual strength measurement (MRC 3–4/5), while lung function remained stable.

**Fig. 3:**
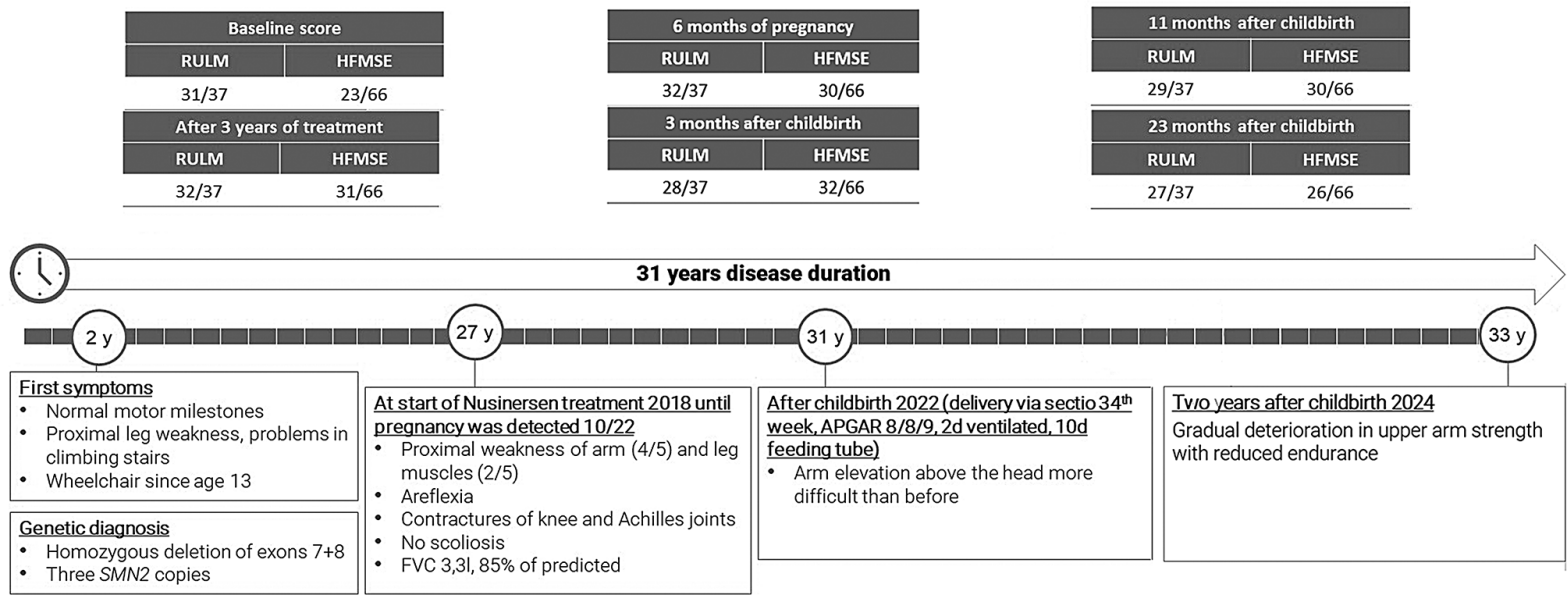
Timeline of our patients’ history

Four months after restarting therapy, RULM started to show slight improvement (from 28 to 29 points bilaterally). In the subsequent assessments over two years, the values for HFMSE and RULM stagnated at a lower level compared to pre-pregnancy values (HFMSE 26 to 27 points, RULM 25 to 29 points). Subjectively, the patient reported a gradual deterioration in upper arm strength with reduced endurance. Figure 3 summarizes the most important milestones of our patients’ history, including the relevant physiotherapeutic assessments.

## Discussion

Due to improved therapeutic options for spinal muscular atrophy, more patients reach childbearing age and consider parenthood and pregnancy. Therefore, pregnancy and family planning will become an increasingly important topic for SMA patients.

So far, only limited data are available to help guide neurologists or obstetricians in counselling their SMA patients on issues related to pregnancy and childbirth. Most of the available data are anecdotal and based on smaller case series in genetically heterogeneous SMA populations from the pre-treatment era. Few existing studies have shown positive maternal and fetal outcome in the majority of patients [8]. The study by Rudnik et al. in 1992 showed positive overall outcome in twelve patients, although muscle weakness increased in the majority of patients during pregnancy. 35% of the patients even showed permanent deterioration of muscle strength. Other complications consisted of an increased rate of necessary caesarean sections, premature and prolonged labour along with prolonged postpartum recovery. Similar complications have also been shown in other studies [21], as well as an increased rate of urinary tract infections [22] and a deterioration in respiratory function [7, 23]. Pregnant SMA patients should be closely supervised by a multi-professional team (gynecologists and neurologists specialized in neuromuscular diseases) to identify possible complications at an early stage and provide the best possible treatment. Our patient developed HELLP syndrome (hemolysis, elevated liver enzymes and thrombocytopenia) during pregnancy. HELLP syndrome was not mentioned as a complication in former studies of SMA patients, and can be seen as a coincidence in this patient. In 2017, a cross-sectional questionnaire-based study of women with SMA in the USA (*N* = 32; type 2 and 3) reported positive pregnancy experiences and outcomes but concluded that caution should be exercised during counselling due to intermittent or permanent worsening of muscle strength and ambulation in the majority of patients following pregnancy [24]. However, 91% were happy with their decision for a child and would do it again, and 85% were satisfied with their obstetric and anesthetic care. Out of 35 pregnancies, there were no significant differences regarding birth weight or APGAR, but more premature births and cesarian sections versus the reference population, especially in SMA2 patients. A recent literature review identified 100 reported pregnancies in 67 patients with SMA (*N* = 67; type 2, 3 and 4) with varied disease characteristics, and found that pregnant patients with SMA faced challenges, yet their overall outcomes and experiences were positive in most cases [7]. However, transient or permanent worsening of motor function was seen in 42% of patients. Interestingly, SMA patients had no fertility problems and no post-partum depression; in wheelchair-bound patients, worsening of respiratory function during pregnancy was seen. There was an increased risk for anesthesia in patients with impaired lung function, and an increased postoperative risk for muscle weakness and respiratory insufficiency. Out of 100 pregnancies, there was no increase in frequency of fetal complications versus the reference population, but frequent preterm birth in Week 36 of pregnancy, and higher rates for delivery via cesarian section, forceps or vacuum delivery, predominantly in non-ambulant patients.

To date, there is only very limited experience with the use of Nusinersen in pregnant women. According to the Information for healthcare professionals, animal studies have not shown any evidence of direct or indirect adverse health effects regarding reproductive toxicity. Nusinersen does not cross the blood-brain barrier to any relevant extent, but for precautionary reasons, the use of Nusinersen should be avoided during pregnancy and breastfeeding [25]. Nusinersen is a non-placental drug [25] showing no harmful effect on embryonic and fetal development in animal studies [26].

Interestingly, there is a first short report from Poland, briefly describing successful pregnancy outcome with start of Nusinersen treatment during late pregnancy [17]. In this 21-year-old ambulant patient, SMA was only diagnosed during the 2nd trimester of pregnancy, and Nusinersen therapy was then started in the 3rd trimester due to worsening of her walking ability, after individual case approval from the coordinating team for the treatment of SMA patients, the Ministry of Health, and the National Health Fund in Poland. However, the reported data are yet too scarce for changing the general recommendations on Nusinersen treatment during pregnancy.

In contrast, there is a fertility risk for Risdiplam – the alternate DMT for adult SMA patients - in men and teratogenicity in women; a Risdiplam break of at least four months before conception is recommended according to the information for healthcare professionals; strategies for maintaining fertility (sperm asservation) should be discussed with male patients of reproductive age before starting treatment. In women, attention is drawn to possible embryofetal toxicity, which has been shown in animal studies; a therapy break of at least one month is recommended before pregnancy occurs. Safe contraception is generally obligatory with Risdiplam; the pregnancy status of patients capable of reproduction should be checked before starting treatment with Risdiplam. It is also recommended not to breastfeed during treatment with Risdiplam [20].

There is some published experience with pregnancies that have occurred during treatment with Nusinersen [16, 17]. Therapy should be started after a Nusinersen break due to pregnancy/breastfeeding in accordance with the dosing regimen specified in the Nusinersen Information for healthcare professionals [25] if the dose is delayed or omitted; pharmacokinetic studies have shown that an additional dose should be given for each omitted maintenance dose to achieve steady-state exposure in the cerebrospinal fluid [19]. Accordingly, our patient received two additional loading doses of Nusinersen at a 14-day interval at therapy re-initiation, as two doses were missed due to pregnancy. Afterwards, administration returned to 4-month intervals according to the usual schedule.

The desire to have children is increasingly getting into the focus of patients, against this background competent and taboo-free counselling is of great importance.

During pregnancy, our patient developed high blood pressure and HELLP syndrome leading to premature caesarean section in the 34th gestational week. However, mother and child were well afterwards, without prolonged postpartum recovery. The patient’s muscle strength, particularly in the upper extremities, decreased during pregnancy and stabilized at a lower level despite restarting Nusinersen therapy only six weeks after the expected gestational date. It remains to be discussed whether the deterioration was primarily due to pregnancy, which would correlate with the findings of previous studies [7, 8, 16, 24], or whether it was due to the natural course of the disease. However, discontinuation of Nusinersen during pregnancy and breast-feeding additionally contributed to declining motor functions.

A case report by Schön et al. 2023 on pregnancy and Nusinersen reported on a 36-year-old patient with SMA3 who was treated with Nusinersen up to twelve weeks until pregnancy was detected. The patient was expecting twins, but one fetus never developed due to fetal growth retardation - an effect that was not attributed to Nusinersen due to the completely normal development of the second fetus [16]. Normal fetal development was always present in our patient, while the last dosage of Nusinersen was administered in the third week of pregnancy.

Similar to our patient, motor function of the patient in Schön et al. [16] deteriorated after discontinuation of Nusinersen – compared to real-world Nusinersen data in adult patients, it can be considered as certain that treatment with Nusinersen improves motor function in longstanding disease even in clinically advanced stages; however, after discontinuation of treatment, further progression mirroring the natural history of the disease is anticipated [27–31]. Compared to pre-treatment era data of pregnant SMA patients, worsening of muscle function in our patient and in the patient of Schön et al. [16] is partly due to the natural course of the disease without SMN restoring treatment, and partly to well-known accelerated worsening during pregnancy in SMA patients [7, 8, 16, 24].

Overall, the patient of Schön et al. showed “stronger” baseline score levels (HFMSE 50, RULM 37), with a ceiling effect in RULM and preserved ambulation, whereas our patient had already been dependent on a wheelchair for decades. Similar to our patient, the patient of Schön et al. showed deterioration in HFMSE after discontinuation of therapy (60 to 55 points), although not falling back to baseline values (50 points).

RULM values deteriorated compared to baseline in our patient (30 to 28 points), although Nusinersen was reinitiated only three months after delivery. In contrast, the patient of Schön et al. showed stable values for RULM (37 points baseline, 36 points 12 months after therapy interruption) and did not restart Nusinersen therapy [16].

Overall, SMA patients should be thoroughly informed about the possibilities and risks of a pregnancy at an early stage. As a best-case scenario, patients should be encouraged to consider a pregnancy when motor functions and respiratory functions are still broadly retained. Although cases of SMA type 2 patients completing their pregnancy with good outcome are reported [32, 33], pregnancy in these patients should be considered as early as possible in order to avoid additional complications. However, our case shows that pregnancy with good maternal and fetal outcome is also possible in severely affected patients with wheelchair dependency. In the light of to-date available animal studies and real-world patient data (our patient [15, 16, 18]), it seems considerably safe to counsel patients to only stop Nusinersen after a pregnancy is detected for the duration of pregnancy and breast-feeding, but not prior to getting pregnant as a precautionary measurement, to avoid prolonging the non-treatment interval and thereby risking irreversible worsening.

Future real-world data will hopefully show whether it is safe continuing treatment with Nusinersen during pregnancy in order to maintain muscle function and to prevent worsening.

Importantly, “family planning” should be a central aspect in the counselling of adolescent and adult patients, especially regarding the choice of DMT in patients with the desire to have children. The wish for parenthood is strongly increasing within the SMA community, highlighting the high unmet need for competent advice.

## Data Availability

No datasets were generated or analysed during the current study.
